# Evaluating Cardio-Protective Molecules by Efficacy Based on Weight Reduction and HbA1c Targets

**DOI:** 10.3390/biomedicines13092306

**Published:** 2025-09-20

**Authors:** Teodor Salmen, Valeria-Anca Pietrosel, Flaviana-Veronica Urzica, Diana-Elena Visan, Bianca-Margareta Salmen, Radu-Cristian Cimpeanu, Delia Reurean-Pintilei, Mihaela-Simona Popoviciu, Anca Pantea Stoian

**Affiliations:** 1 Doctoral School, Carol Davila University of Medicine and Pharmacy, 020021 Bucharest, Romania; teodor.salmen@drd.umfcd.ro (T.S.);; 2 Pitesti Emergency County Hospital, 110283 Pitesti, Romania; 3 DiabetMed Clinic, 052034 Bucharest, Romania; 4 N.C. Paulescu National Institute of Diabetes, Nutrition and Metabolic Diseases, 030167 Bucharest, Romania; flaviana-veronica.urzica@rez.umfcd.ro (F.-V.U.); diana-elena.visan@rez.umfcd.ro (D.-E.V.); 5 Doctoral School, University of Medicine and Pharmacy of Craiova, 200349 Craiova, Romania; 6 Department of Medical-Surgical and Complementary Sciences, Faculty of Medicine and Biological Sciences, “Ștefan cel Mare” University, 720229 Suceava, Romania; delia.pintilei@usm.ro; 7 Faculty of Medicine and Pharmacy, University of Oradea, 410073 Oradea, Romania; 8 Department of Diabetes, Nutrition and Metabolic Diseases, Carol Davila University of Medicine and Pharmacy, 050474 Bucharest, Romania; anca.stoian@umfcd.ro

**Keywords:** T2DM, SGLT-2i, GLP-1 RAs, weight loss, metabolic control

## Abstract

**Background**: The effectiveness of GLP-1 RAs and SGLT-2i classes, evaluated by a HbA1c target of <7% and body weight reduction (BWR) of 5% in patients with type 2 diabetes mellitus (T2DM), represents the aim of this article. **Methods**: A retrospective analysis was conducted on consecutively admitted out-patients of a tertiary care center for diabetes mellitus (DM) treatment from Romania, where 405 enrolled patients were evaluated at baseline, 6-, and 12-month visits. **Results**: SGLT-2i were superior to GLP-1 RAs and metformin, providing higher rates of combined target achievements—22.7% at 12 months, *p* < 0.001. Both HbA1c reduction and BWR were more consistent at the 12-month visit: 6.58% for metformin, 22.68% for SGLT-2i, and 5.88% for GLP-1 Ras, as compared to baseline while the 12-month visit results were as follows: 4.79% for metformin, 5.04% for SGLT-2i and 5.88% for GLP-1 RAs as compared to the 6-month visit. Despite the fact that the HbA1c < 7% target at baseline was 38.92% for metformin, 17.64% for SGLT-2i, and 41.17% for GLP-1 RAs, BWR was achieved less, probably influenced by insulin treatment. **Conclusions**: This study shows real-world Romanian efficacy and over the time response of administering the new classes in T2DM patients when aiming for HbA1c < 7% levels and 5% BWR, with SGLT-2i outperforming metformin and GLP-1 RAs, emphasizing their growing role in the management of T2DM.

## 1. Introduction

The most significant non-transmissible chronic illnesses currently include cancer, cardiovascular (CV) diseases, chronic respiratory diseases and type 2 diabetes mellitus (T2DM) which constitute the modern-day pandemics in this category [1]. Obesity and T2DM alongside their associated comorbidities represent a severe burden on healthcare systems, constituting 6% and 3–12%, respectively, of the total healthcare systems’ budget, depending on the financial management of each country [2,3].

The available data suggests an alarmingly increasing incidence of T2DM and obesity, while in Southeastern Europe, including Romania, approximately 80% of the population has a body mass index (BMI) > 25 kg/m^2^, and a prevalence of obesity of 32.1% [4].

The PREDATORR study was conducted in Romania in 2014, reporting a prevalence of 11.6% for T2DM and 16.5% for prediabetes in 2014 [5]. According to the National Institute of Public Health from Romania, from 2012 to 2021, the incidence of DM increased by 50.72% [6]. Furthermore, projections suggest that by 2035, 50% of the world’s population will be affected by obesity, and 55% will be diagnosed with T2DM [5,7].

Obesity, T2DM management alongside with their comorbidities, respectively, CV diseases (CVD), angina pectoris, heart failure (HF), chronic kidney disease (CKD), and conditions such as high blood pressure (BP) and dyslipidemia, through prescriptions and inpatient procedures, receive significant allocations in Romania, that surpass those of many other countries in Southeastern Europe [4,8].

Body weight reduction (BWR) stands out as one of the most effective tools in treating T2DM, particularly if associated with obesity and/or CVD. However, achieving BWR is challenging due to the need for major lifestyle adjustments, until the emergence of glucagon-like peptide-1 receptor agonist (GLP-1RAs) and sodium-glucose cotransporter-2 inhibitors (SGLT-2i), that are efficient in BWR, as well as in improving metabolic and glycemic control, with cardio–renal protection, by significantly reducing the occurrence of major adverse CV events (MACE), and HF-related incidents [8,9,10,11].

SGLT-2i facilitates the urinary excretion of glucose, reducing HbA1c, normalizing fasting plasma glucose (FPG), and improving postprandial glucose levels, with additional calorie loss that leads to gradual BWR [12,13,14]. Clinical trials have demonstrated that SGLT-2i induce modest yet notable decreases in both systolic and diastolic BP, enhance metabolic control by ameliorating triglyceride (TG) levels and increasing high-density lipoprotein-cholesterol (HDL-C) levels, consequently reducing overall CV risk [15,16,17,18]. In patients with T2DM and established CVD, SGLT-2i minimizes the risk of MACE, including CV death, non-fatal myocardial infarction, and non-fatal stroke. Trials have also demonstrated the efficacy of this medication in reducing the risk of CV hospitalization and death for HF, initially in cases with HF with reduced ejection fraction (EF), and more recently on HF with mildly reduced or preserved EF [19,20]. Moreover, SGLT-2i proved to slow the progression of CKD, reduce albuminuria, preserve kidney function in T2DM patients and also in those without pre-existing kidney disease [21]. These data are scarcely evaluated in real-world Romanian studies.

GLP-1 RAs mimic the function of natural GLP-1 by enhancing insulin secretion in response to glucose and inhibiting excessive glucagon secretion, leading to a decrease in HbA1c, FPG, and postprandial glucose excursions, all without the risk of hypoglycemia [22]. The changes in weight, BMI, and HbA1c reduce systolic BP (SBP) [23]. Clinical trials, including LEADER, SUSTAIN-6, and REWIND, have shown that specific GLP-1 RAs can lower the risk of MACE and slow the progression of CKD by improving outcomes related to albuminuria; data that are scarcely evaluated by real-world Romanian studies [24]. GLP-1 RAs contribute to BWR through a neural pathway that directly affects appetite, leading to decreased food intake, also inhibiting postprandial gastric emptying and reducing gastric secretion, while promoting thermogenesis [25].

HbA1c targets should be individualized by taking into consideration the disease duration of evolution, comorbidities, age or other factors that deter frailty, but the majority of guidelines recommend that 6.5% should not be strict and allow the value of 7% as a more realistic target [26,27].

Reduction in excess weight significantly enhances glycemic control, for example, a reduction of 15% or more has the potential to fundamentally alter the course of the disease, with documented instances of remission, while attaining a BWR of 5 to 10% is recognized for its positive impact on both metabolic and glycemic outcomes in individuals with T2DM [28]. Even if the guidelines recommend various specific approaches to weight loss—from reducing a specific number of calories per day (e.g., 500–750), or a weight loss of 0.5–1 kg per week, or increasing the amount of physical activity per day—they all agree that at least achieving a weight loss of 5% is recommended [27,29].

Given the fact that the protocols and guidelines are very diverse worldwide and that real-world studies are rare, conducted on small samples, or are conducted following varied protocols, with the last large Romanian study, the PREDATORR trial, performed over a decade ago contributing to the literature with only epidemiologic data regarding Romanian patients, the aim of this research is to assess the effectiveness of GLP-1 RAs and SGLT-2i classes in achieving a BWR exceeding 5% of the initial weight, and maintaining an HbA1c level below 7% in patients with T2DM who are receiving standard T2DM treatment in real-world Romanian clinical practice.

## 2. Materials and Methods

A retrospective, observational evaluation was conducted following the Declaration of Helsinki and approved by the Institutional Ethics Committee of N Paulescu National Institute for Diabetes Mellitus, Nutrition and Metabolic Disorders, Bucharest, Romania (protocol number 5591, from 17 November 2022), but without calculating sample size prior to its approval. It included consecutively admitted patients with T2DM that received treatment in a standard-of-care (depending on the national guidelines, availability, and last but not least, clinical judgment) and in the maximum tolerated regimen, during 2019 at the N Paulescu National Institute for Diabetes Mellitus, Nutrition and Metabolic Disorders’ Outpatient Department. The patients were included if they met the inclusion criteria. Both the inclusion and exclusion criteria are shown in Table 1.

The substances approved by the National Agency for Medicines and Medical Devices of Romania in the national protocol of prescription that were administered to the patients, of the drugs of interest, were empagliflozin and dapagliflozin for SGLT-2i, and dulaglutide, lixisenatide, semaglutide, and exenatide for GLP-1 RA. All patients received treatment in the maximum tolerated dose as recommended by national standard-of-care protocols and maintained it for at least 6 months—partially reflecting their adherence.

The patients’ data were collected from the hospital’s electronic database and included the parameters of interest as follows: demographic elements (e.g., age, gender, and settlement), clinical (height, weight, BMI), comorbidities (e.g., high BP and dyslipidemia, etc.), paraclinical elements (HbA1c), and data about the treatment (antidiabetic, BP-lowering, and lipid-lowering drugs). They were collected at baseline (V0M), at the 6-month visit (V6M), and the 12-month visit (V12M). Furthermore, the data were systematized into Excel tables and analyzed using both Excel and PSPP software 2.0.1 version.

This study is part of a larger doctoral study whose main objectives were to evaluate the efficacy [10] and safety [9] of the molecules of metformin, SGLT-2i, and GLP-1 RAs. In the efficacy study, the lipidic profile (total-cholesterol, HDL-cholesterol, LDL-cholesterol, triglycerides), BMI, heart rate, SBP and diastolic BP, and fasting glycaemia were evaluated [10], while in the safety study, urea, creatinine, urinary albumin-to-creatinine ratio, estimated glomerular filtration rates, GOT and GPT, and adverse reactions were evaluated [9]. Meanwhile, the present study is a secondary analysis that is centered on the efficacy of metabolic control—attainment of a HbA1c < 7% and a BWR of >5%, respectively.

**Figure 1:**
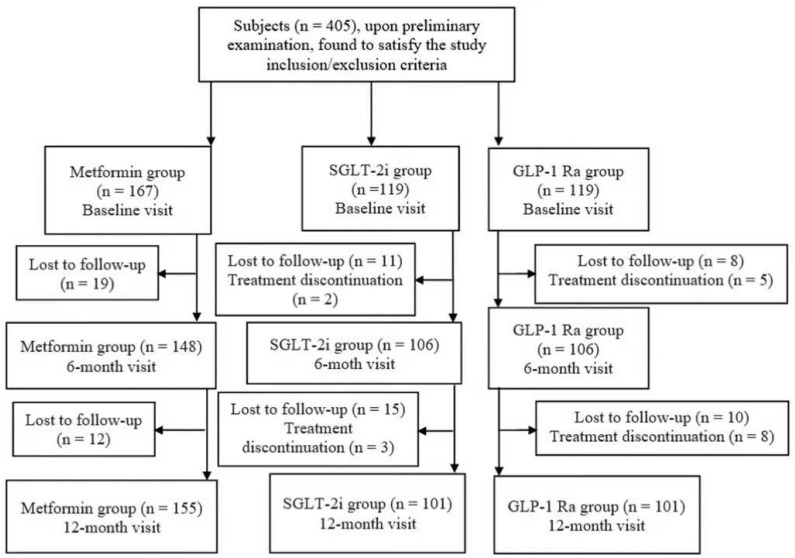
Flowchart of the patients.

Because some of the 405 patients missed either the V6M or the V12M, we performed the last observation carried forward system in order to preserve the sample size and the real-world clinical retention patterns, as seen in the flowchart from Figure 1.

Data processing was performed using PSPP software and Excel 2010 software. For the interest parameters, we calculated mean values, frequency ranges, standard deviations, statistical significance tests using Student’s method (*t*-test), and χ2. ANOVA was used to compare means, and the statistical significance level was 0.05.

## 3. Results

The demographic data of the included patients at the V0M are presented in Table 2.

The comorbidities and DM complications of the included patients at V0M are presented in Table 3.

The patients that achieved HbA1c < 7% at each visit are presented in Table 4.

The patients that achieved HbA1c < 7%, BWR ≥ 5% and both HbA1c < 7% and BWR ≥ 5% between the visit of interest are presented in Table 5.

The comparisons for HbA1c < 7%, BWR ≥ 5% and both HbA1c < 7% and BWR≥5% between treatment groups and between follow-up visits are presented in Table 6.

At baseline, the metformin group was younger, with a shorter duration of DM, while the SGLT-2i group had higher HbA1c and BMI, while also being predominantly males. HBP is the most frequent comorbidity and, also, is more prevalent in SGLT-2i groups. The GLP-1 RAs group predominantly has polyneuropathy and CKD, and received insulin in the greatest percentage.

At follow-ups, all groups revealed glycemic control improvement, with SGLT-2i having the most powerful effect, with more than half of the patients obtaining HbA1c values < 7% and BWR > 5% at the V12M.

So, the most efficient in reducing both HbA1c < 7% and obtaining a BWR > 5% in this real-life study were SGLT-2i, compared to metformin and GLP-1 RAs.

## 4. Discussion

This study aimed to evaluate the efficacy of novel antidiabetic non-insulin drugs, respectively, SGLT-2i, and GLP-1 RA versus metformin, in reaching an HbA1c < 7 % and a BWR of at least 5%, in patients with T2DM in a real-life setting. The most efficient in reducing both HbA1c < 7% and obtaining a BWR > 5% in this real-life study were SGLT-2i (22.68% of cases, *p*-value < 0.001 vs. metformin, *p*-value < 0.001 vs. GLP-1 RA). None of the included patients, receiving either GLP-1 RA, SGLT-2i, or metformin, met the cumulated endpoint of achieving an HbA1c < 7% and at least 5% BWR between the first two visits. However, between the V0M and V12M, up to 22.68% patients treated with SGLT-2i, 6.58% patients treated with metformin, and 5.88% patients treated with GLP-1 RA achieved the cumulated goal. Most of the HbA1c decrease and the BWR was obtained at the V6M and V12M.

In our study, HbA1c < 7% was present at the V0M in almost half of the patients treated with GLP1-RA (41.17%) and only in 17.64% of the patients with SGLT-2i, while at the V6M, almost half of the patients in each group achieved this result (50.29% treated with metformin, 52.1% treated with SGLT2i, 53.78% treated with GLP1-RA); and at V12M, the results are similar (49.1% with metformin, 51.26% with SGLT2i, and 51.26% with GLP-1 RA), with the small decrease explained by the patients lost to follow-up. Although GLP-1 RA or SGLT-2i combined with metformin have increased potency in reducing HbA1c, it has to be stated that the patients in the metformin group had lower HbA1c at the V0M, and also a decreased duration of DM.

In a study involving 410 Korean patients that evaluated the efficacy of SGLT-2i after 12 weeks, only 18.3% achieved an HbA1c < 7%, in contrast to the results of our study with a slightly longer period, while more than half of the patients achieved the HbA1c target at the V6M [30]. In the LEAD-6 clinical trial which evaluated liraglutide versus exenatide twice daily, they obtained a statistically significant greater reduction in HbA1C (−1.12% for liraglutide vs. −0.79% for exenatide) with HbA1c < 7% in a larger proportion of patients treated with liraglutide as compared to exenatide (54 vs. 43%) [31]. While comparing exenatide once weekly with exenatide twice daily, the drop in HbA1c was −1.9 [SE 0.1%] vs. −1.5 [0.1%], 95% CI −0.54% to −0.12%; *p* = 0.0023, with a significantly greater proportion of patients in the first group achieving the target of HbA1c ≤ 7%, (77% vs. 61%, *p* = 0.0039) [32]. In the AWARD-1 study, patients receiving dulaglutide 1.5 mg achieved a further 1.5% HbA1c reduction from the V0M mean HbA1c of 8.1%, with 78% achieving the goal of <7.0% [33].

Furthermore, in a meta-analysis by Esposito et al. studying the efficacy of GLP-1RA versus placebo in reducing HbA1c levels, the proportion of the patients who reached the glycemic control was 46% for exenatide, 47% for liraglutide, and 63% for exenatide long-acting release; these are results that are similar to our study: HbA1c < 7% in 53.78% of the patients at the V6M, and 51.26% maintained this level at the V12M [34].

In a systematic review by Young et al. [35] that thoroughly searched for the heterogeneity of GLP-1 RA or SGLT-2i efficacy in reducing HbA1c or on CV outcomes, the authors highlighted that the higher the HbA1c at V0M, the greater the reduction with both SGLT-2i and GLP-1 Ras—similar to our study. However, the authors did not find any studies comparing the relative efficacy of GLP1-Ra vs. SGLT-2i in relation with baseline HbA1c. Moreover, they studied different factors that can influence the glycemic reduction effect. For instance, SGLT-2i showed that HbA1c reduction is substantially altered with lower eGFR. On the other side, when it comes to the effect of GLP1-RAs, the authors followed the markers of reduced β-cell function (such as longer DM duration [or proxies such as insulin treatment], diminished fasting C-peptide, lower urine C-peptide-to-creatinine ratio, positive antibodies to glutamic acid decarboxylase or islet autoantibodies type 2), which were associated with lesser glycemic response to GLP1-RA in many observational studies. However, the post hoc analysis of the randomized controlled studies failed to confirm this connection. No association between other clinical features such as BMI, gender, age, kidney function, and insulin resistance markers were found [35]. Another proposed theory for reduced overtime effectiveness would be the appearance of anti-drug antibodies. However, there are not enough studies to confirm this affirmation, and it was not proven that this could have an impact on HbA1c [36,37]. SGLT-2i are independent from the β-cell function or insulin sensitivity [38], and more recent studies on animal models showed that dapagliflozin promotes β-cell regeneration by inducing pancreatic endocrine cell phenotype conversion in mice with T2DM. More studies are needed in order to better characterize the efficacy heterogeneity of novel antidiabetic classes in order to properly choose between molecules.

Overall, in our cohort, SGLT-2i performed better in lowering HbA1c than metformin alone, and then GLP-1 RA as well (*p* < 0.001 in both comparisons). Only 19.32% of the patients decreased their A1c below 7% after one year of GLP-1 RA administration versus 39.49% with SGLT-2i, and 19.16% with metformin, but we highlight the fact that almost 40% of the patients were well-controlled at the V0M in the GLP1-RA group and in the metformin group, compared to only 17.64% in the SGLT-2i group. Another interesting fact we observed is the high percentage of patients requiring insulin in the GLP-1 RA group, which usually comprises patients who have difficulty in achieving the HbA1c target. On the contrary, patients that received only metformin had a smaller duration of DM (median of 3 years), and could have been closer to the debut of the disease, where the HbA1c is greatly impacted by the adherence to diet and metformin initiation.

SGLT-2i achieved most frequently the 5% BWR, with 52.1% of patients at the V6M vs. 15.12% in the GLP-1 RA group, and only 12.57% in the metformin group (*p* < 0.001 in both cases). When comparing the V12M and the V0M, the percentage slightly increased, up to 53.78% for SGLT-2i, 20.16% for GLP1-RA, and 17.36% for metformin, with significant difference for the gliflozins (*p* < 0.001 in both cases). The BWR of 5% was partly achieved during the first 6 months of monitoring, with only few patients managing to lose weight from the V6M to the V12M. It is intriguing to explain why the vast majority achieved this BWR during the first 6 months—possibly due to a stronger motivation and adherence to associated lifestyle recommendations, which unfortunately is hard to quantify, or there could be another cause, which requires further research.

SGLT-2i lower glycaemia in an insulin-independent mechanism, while the urinary excretion of glucose, hence leading to weight loss, improved blood glucose levels, insulin-sensitivity, and an increased fasting and post-meal glucagon level. Furthermore, it shifts the energy substrate from carbohydrates to lipid storages which are mobilized, diminishing glucose oxidation while lipolysis is accelerated with increased fatty acid oxidation and the increased production of ketone bodies, similar to the fasting state, which cause fat mass and weight loss in the long run [13,18]. In a randomized controlled trial, 10 mg of dapagliflozin showed a BWR over 2 years of treatment, with a faster decline over the first few weeks followed by a more gradual decline to the 24th week that had not plateaued by week 102 [39]. However, adaptive mechanisms can appear through an increase in appetite or caloric intake, hindering the BWR in time [18]. In this direction, a study on Asian subjects with T2DM treated with SGLT-2i showed that approximately half (45.6%) of the patients achieved a significant BWR (defined as BWR >3% in 1 year) with an intriguing association with age over 70 years [39,40].

Several trials showed important BWR when using GLP-1 RAs. For example, liraglutide as compared to the placebo, evaluated in the SCALE Diabetes trial, induced a BWR ≥ 5% in 40.4% of patients with liraglutide (1.8 mg—approved for T2DM) vs. 21.4% (estimated difference for liraglutide [1.8 mg] vs. placebo, 19.0% [95% CI, 9.1% to 28.8%]; *p* < 0.001). BWR ≥ 10% occurred in 15.9% with liraglutide (1.8 mg) vs. 6.7% with placebo (estimated difference for liraglutide [1.8 mg] vs. placebo, 9.3% [95% CI, 2.7% to 15.8%], *p* = 0.006) [41]. Furthermore, in a SCALE Insulin trial, liraglutide 3.0 mg achieved a mean BWR of 5.8%, versus −1.5% with placebo (estimated treatment difference −4.3% [95% CI -5.5; −3.2]; *p* < 0.0001), with 51.8% of individuals having a BWR ≥ 5% versus 24.0% with placebo (odds ratio 3.41 [95% CI 2.19; 5.31]; *p* < 0.0001), in T2DM patients associating insulin treatment [42]; however, liraglutide is approved at a maximum 1.8 mg dose for DM. Semaglutide efficiency in BWR was evaluated in both doses of 1 mg (approved for T2DM) and 2.4 mg (approved for obesity) in a STEP-2 trial, and 68.8% of subjects achieved a BWR ≥ 5% from the V0M, but insulin was notably not used in patients [43].

A real-world study among 589 patients initiating a GLP-1 RA, with a median BMI of 41.2 kg/m^2^ (IQR (35.8, 46.4)) showed that only a minority of subjects reached a BWR ≥ 5% of baseline weight, 33.4% and 43.5% at 12 and 24 months, respectively, with a part of them having reduced adherence over time, 64.5% and 59.2% at 12 and 24 months, with discontinuation occurring in 45.2% and 64.7% of the cases, respectively [44].

As for the little BWR in the GLP-1 RA group in our study, a possible explanation that resides in the statistics is the high number of insulin-treated patients. As Jensterle et al. stated [45], although other possible predictors of weight loss have been studied (e.g., baseline BMI, BMI change at 1 month, incidence of nausea and vomiting, delayed gastric emptying shortly after intervention, baseline appetite and satiety measures, and some polymorphisms in the GLP-1 receptor), to date, there are no reliable predictive models to assess the individual BWR potential, and it is considered that the inter-individual variability regarding weight loss is greater than that for glycemic control [46]. What should not be forgotten is that other factors can modify the response to GLP-1 RAs, such as associated medications/diseases, altered microbiota, genetic and socio-familial background, adherence/motivation to lifestyle changes or fear of hypoglycemia (higher in people treated with insulin or sulfonylurea, but not mandatory), and possible occurrence of GLP-1 RAs adverse effects which would rend the patient to improperly administer the medication. Tachyphylaxis or the inability to intensify lifestyle modifications when reaching the plateau is also discussed. Last but not least, we recall the tendency of the organism to go back to the initial weight due to the reactivation of hunger hormones such as ghrelin, and also by slowing down the metabolism through the lack of increased physical activity, as lean body mass is also lost.

Furthermore, we remind ourselves again that very few patients received more potent GLP-1 RAs such as semaglutide. In a meta-analysis of Hong et al., including 61 RCTs and 17281 participants, both GLP-1 RA and SGLT-2i conferred “similar” BWR, but by far to a greater extent for semaglutide 2.4 mg with results comparable to semaglutide 1.0 mg, as opposed to the STEP-2 trial who favored the higher dose. Both doses of semaglutide were associated with a BWR of more than 5 kg, while the result of semaglutide 1.0 mg was with evidence of low certainty. In Romania, the maximum administered dose approved for TD2M is 1 mg. Liraglutide 1.8 mg, exenatide 10 µg, dulaglutide 1.5 mg, and dapagliflozin 10 mg reduced body weight from −3.14 kg to −1.28 kg. Canagliflozin 300 mg and empagliflozin 10 mg had modest effects in BWR. As for the HbA1c reduction, GLP-1 RA was superior to SGLT-2i (MD: −0.39%, 95% CI −0.70 to −0.08), although with more adverse reactions [47,48,49].

The combination of SGLT-2i with GLP1-RA that has different mechanisms of action offers greater benefits in lowering CV risk, blood glucose levels, and promoting BWR [50]; however, we did not include patients that receive both medications, as local protocols did not allow such a combination at that time.

In the future, a more in-depth understanding of heterogeneous treatment efficiency based on patients’ characteristics could increase the capacity to predict individual responses to medication and choose the most suitable option as part of precision medicine in T2DM [51].

The limitations of our study are the relatively small number of patients enrolled, the relatively short follow-up period, the missing patients at follow-ups, the retrospective design of the study with a reliance on patient records without the possibility to quantify the degree of treatment, diet, physical activity adherence, and also the lack of data to analyze the adherence, safety, and therapy discontinuations. Also, a high percentage of insulin-treated patients in the GLP-1 RAs group could be a cofounder, and the last observation carried forward may introduce bias, especially if the missingness is not random, but rather missingness is below 10% at each time point and no other imputation methods were applied, making the results potentially biased by attrition.

More data is needed to provide a better perspective on the efficacy of SGLT-2i and GLP-1 RAs in BWR and HbA1c reduction in real-life cases. Further prospective studies should be carried out including larger and heterogeneous populations (ethnicities, demographics, biological characteristics). Possibly, body composition determination would be a useful resource for better understanding the underlying mechanisms leading to different levels of efficacy for these particular medications, alongside medication adherence and discontinuation, which are critical for real-world effectiveness.

On the other hand, the strength of this study lies in the fact that the data from real-life Romanian practice are scarcely reported. Although it enrolled a limited number of patients, it sums up the few pre-existing studies in the literature which also did not benefit from significant cohorts.

## 5. Conclusions

This study shows a sample of patients representative for the Eastern European population, and the impact of the treatment with SGLT-2i and GLP-1 RAs in real-life practice, that can be added to the current literature, emphasizing the real-world efficacy and response over time of administering new classes to T2DM patients regarding glycemic and weight reduction. In this real-world study, SGLT-2i were the most effective as compared to metformin and GLP-1 Ras, both in glycemic control with HbA1c < 7% and BWR ≥ 5%.

## Figures and Tables

**Table 1:** Inclusion and exclusion criteria.

Inclusion Criteria	Exclusion Criteria
Adults over 18 years old	Younger than 18 years old
Positive diagnosis of T2DM with a duration of at least six months	Type 1 DM, secondary DM, other types of DM
Standard-of-care treatment for T2DM with maximum tolerated doses	Presence of any disease that can be associated with involuntary weight reduction
Minimum 6 month-treatment with SGLT-2i and GLP-1 RAs prior to the baseline visit	Presence of anemia (defined as lower hemoglobin than the laboratory inferior threshold)/hemoglobinopathies or other causes that can interfere with hemoglobin A1c assay

DM—diabetes mellitus; T2DM—type 2 diabetes mellitus; SGLT-2i—sodium-glucose cotransporter-2 inhibitors; GLP-1 RA—glucagon-like peptide 1 receptor agonist.

**Table 2:** Demographic characteristics of the included patients.

	Metformin (n = 167)	SGLT-2i (n = 119)	GLP-1 RA (n = 119)
Males, n, %	65 (38.92%)	85 (71.42%)	60 (50.42%)
Age (years), mean, SD	57.95 ± 10.1	56.89 ± 10.51	59.26 ± 9.09
DM duration (years), median, (IQR)	3 (1, 10)	7, (4, 12)	8 (4, 13)
HbA1c (%)	7.39 ± 1.28	8.12 ± 1.65	7.42 ± 1.43
Body weight (kg), mean, SD	93.73 ± 19.05	100.67 ± 19.56	92.57 ± 16.68
BMI (kg/m^2^), mean, SD	31.83 ± 5.81	35.54 ± 6.52	32.15 ± 6.15

SGLT-2i—sodium-glucose cotransporter-2 inhibitors; GLP-1 RA—glucagon-like peptide-1 receptor agonists; IQR—interquartile range; SD—standard deviation; DM—diabetes mellitus; BMI—body mass index.

**Table 3:** Comorbidities and T2DM complications of the included patients at baseline.

	Metformin (n = 167)	SGLT-2i (n = 119)	GLP-1 RA (n = 119)
HBP	139 (83.2%)	111 (93.3%)	107 (89.9%)
ASCVD	46 (27.5%)	51 (42.9%)	42 (35.3%)
HF	10 (5.9%)	14 (11.8%)	8 (6.7%)
Atrial Fibrillation	3 (1.8%)	8 (6.7%)	3 (2.5%)
PAD	55 (32.9%)	50 (42%)	38 (31.9%)
Amputation	5 (3%)	7 (5.9%)	1 (0.8%)
Polyneuropathy	83 (49.7%)	61 (51.3%)	71 (59.7%)
Vegetative Neuropathy	1 (0.6%)	5 (4.2%)	3 (2.5%)
Retinopathy *	11 (6.6%)	13 (10.9%)	14 (11.8%)
CKD	14 (8.4%)	15 (12.6%)	20 (16.8%)
Need for insulin therapy (%)	16 (9.58%)	15 (12.6%)	61 (51.3%)

SGLT-2i—sodium-glucose cotransporter-2 inhibitors; GLP-1 RA—glucagon-like peptide-1 receptor agonists; HBP—high blood pressure; ASCVD—atherosclerotic cardiovascular disease; HF—heart failure; PAD—peripheral artery disease; CKD—chronic kidney disease; *—GLP-1 RAs were not administered in cases with diagnosed severe preproliferative retinopathy, proliferative retinopathy, or diabetic maculopathy.

**Table 4:** Patients that achieved HbA1c < 7% at each visit.

HbA1c < 7%	V0M, n, %	V6M, n, %	V12M, n, %
Metformin (n = 167)	65 (38.92%)	84 (50.29%)	82 (49.1%)
SGLT-2i (n = 119)	21 (17.64%)	62 (52.1%)	61 (51.26%)
GLP-1 RA (n = 119)	49 (41.17%)	64 (53.78%)	61(51.26%)
SGLT-2i versus metformin	*p* < 0.001	*p* = 0.764	*p* = 0.719
GLP-1 RA versus metformin	*p* = 0.701	*p* = 0.561	*p* = 0.719
SGLT-2i versus GLP-1 RA	*p* < 0.001	*p* = 0.795	*p* = 1

SGLT-2i—sodium-glucose cotransporter-2 inhibitors; GLP-1 RA—glucagon-like peptide receptor agonists; V0M—baseline visit; V6M—6-month visit; V12M—12-month visit.

**Table 5:** Patients that achieved HbA1c < 7%, BWR ≥ 5% and both HbA1c < 7% and BWR ≥ 5% between the visits of interest.

Treatment Group	V6M vs. V0M, n, %	V12M vs. V0M, n, %	V12M vs. V6M, n, %
*HbA1c < 7%*			
Metformin (n = 167)	32 (19.16%)	32 (19.16%)	8 (4.79%)
SGLT-2i (n = 119)	49 (41.17%)	47 (39.49%)	6 (5.04%)
GLP-1 RA (n = 119)	21 (17.64%)	23 (19.32%)	7 (5.88%)
*BWR ≥ 5%*			
Metformin (n = 167)	21 (12.57%)	29 (17.36%)	9 (5.38%)
SGLT-2i (n = 119)	62 (52.1%)	64 (53.78%)	7 (5.88%)
GLP-1 RA (n = 119)	18 (15.12%)	24 (20.16%)	3 (2.52%)
*HbA1c < 7% and BWR ≥ 5%*			
Metformin (n = 167)	0	11 (6.58%)	8 (4.79%)
SGLT-2i (n = 119)	0	27 (22.68%)	6 (5.04%)
GLP-1 RA (n = 119)	0	7 (5.88%)	7 (5.88%)

SGLT-2i—sodium-glucose cotransporter-2 inhibitors; GLP-1 RA—glucagon-like peptide receptor agonists; V0M—baseline visit; V6M—6-month visit; V12M—12-month visit; BWR—body weight reduction.

**Table 6:** Comparisons for HbA1c < 7%, BWR ≥ 5% and both HbA1c < 7% and BWR ≥ 5% between treatment groups and between visits of interest.

Treatment Group Comparison	V6M vs. V0M, n, %	V12M vs. V0M, n, %	V12M vs. V6M, n, %
*HbA1c < 7%*			
SGLT-2i vs. metformin	*p* < 0.001	*p* < 0.001	*p* = 0.923
GLP-1 RA vs. metformin	*p* = 0.745	*p* = 0.972	*p* = 0.718
SGLT-2i vs. GLP-1 Ra	*p* < 0.001	*p* = 0.001	*p* = 0.775
*BWR ≥ 5%*			
SGLT-2i vs. metformin	*p* < 0.001	*p* < 0.001	*p* = 0.858
GLP-1 RA vs. metformin	*p* = 0.535	*p* = 0.548	*p* = 0.233
SGLT-2i vs. GLP-1 Ra	*p* < 0.001	*p* < 0.001	*p* = 0.196
*HbA1c < 7% and BWR ≥ 5%*			
SGLT-2i vs. metformin	-	*p* < 0.001	*p* = 0.923
GLP-1 RA vs. metformin	-	*p* = 0.809	*p* = 0.811
SGLT-2i vs GLP-1 Ra	-	*p* < 0.001	*p* = 0.775

BWR—body weight reduction; SGLT-2i—sodium-glucose cotransporter-2 inhibitors; GLP-1 RA—glucagon-like peptide receptor agonists; V0M—baseline visit; V6M—6-month visit; V12M—12-month visit.

## Data Availability

Data is available on request from the corresponding authors, due to the fact that this is an ongoing doctoral project where data is being analyzed for further publication.

## Abbreviations

The following abbreviations are used in this manuscript:

BMI	Body Mass Index
BP	Blood Pressure
BWR	Body Weight Reduction
CKD	Chronic Kidney Disease
CV	Cardiovascular
CVD	Cardiovascular Disease
DM	Diabetes Mellitus
EF	Ejection Fraction
FPG	Fasting Plasma Glucose
GLP-1 RAs	Glucagon-like Peptide-1 Receptor Agonists
HDL-C	High-density Lipoprotein-cholesterol
HF	Heart Failure
MACE	Major Adverse Cardiovascular Events
SBP	Systolic Blood Pressure
SGLT-2i	Sodium-glucose Cotransporter-2 Inhibitors
T2DM	Type 2 Diabetes Mellitus
TG	Triglyceride
V0M	Baseline visit
V6M	6-month visit
V12M	12-month visit
